# Using the Technology Acceptance Model to Explore User Experience, Intent to Use, and Use Behavior of a Patient Portal Among Older Adults With Multiple Chronic Conditions: Descriptive Qualitative Study

**DOI:** 10.2196/11604

**Published:** 2019-04-08

**Authors:** Jennifer Dickman Portz, Elizabeth A Bayliss, Sheana Bull, Rebecca S Boxer, David B Bekelman, Kathy Gleason, Sara Czaja

**Affiliations:** 1 Division of General Internal Medicine School of Medicine University of Colorado Aurora, CO United States; 2 Institute for Health Research Kaiser Permanente Colorado Aurora, CO United States; 3 Department of Family Medicine School of Medicine University of Colorado Aurora, CO United States; 4 mHealth Impact Lab Colorado School of Public Health University of Colorado Aurora, CO United States; 5 Department of Medicine Eastern Colorado Health Care System Department of Veterans Affairs Denver, CO United States; 6 Division of Geriatrics Weill Cornell Medicine New York, NY United States; 7 Center for Research and Education on Aging and Technology Enhancement University of Miami Miami, FL United States

**Keywords:** multiple chronic conditions, personal health record, patient portals, aging, health information technology

## Abstract

**Background:**

Patient portals offer modern digital tools for older adults with multiple chronic conditions (MCC) to engage in their health management. However, there are barriers to portal adoption among older adults. Understanding portal user interface and user experience (UI and UX) preferences of older adults with MCC may improve the accessibility, acceptability, and adoption of patient portals.

**Objective:**

The aim of this study was to use the Technology Acceptance Model (TAM) as a framework for qualitatively describing the UI and UX, intent to use, and use behaviors among older patients with MCC.

**Methods:**

We carried out a qualitative descriptive study of Kaiser Permanente Colorado’s established patient portal, *My Health Manager.* Older patients (N=24; mean 78.41 (SD 5.4) years) with MCC participated in focus groups. Stratified random sampling was used to maximize age and experience with the portal among participants. The semistructured focus groups used a combination of discussion and think-aloud strategies. A total of 2 coders led the theoretically driven analysis based on the TAM to determine themes related to use behavior, portal usefulness and ease of use, and intent to use.

**Results:**

Portal users commonly used email, pharmacy, and lab results sections of the portal. Although, generally, the portal was seen to be easy to use, simple, and quick, challenges related to log-ins, UI design (color and font), and specific features were identified. Such challenges inhibited participants’ intent to use the portal entirely or specific features. Participants indicated that the portal improved patient-provider communication, saved time and money, and provided relevant health information. Participants intended to use features that were beneficial to their health management and easy to use.

**Conclusions:**

Older adults are interested in using patient portals and are already taking advantage of the features available to them. We have the opportunity to better engage older adults in portal use but need to pay close attention to key considerations promoting usefulness and ease of use.

## Introduction

### Background

Patient portals, also referred to as tethered personal health records, are secure websites for personal health information and patient resources directly linked to a provider’s electronic health record [1,2]. Patient portals offer modern digital tools for older adults to engage in health management and with their health care system [3]. In the United States, patient portals vary greatly by provider and health care system, but often provide access to personal health information, email messaging with providers, appointment schedulers, and prescription management [1]. Patient portals are designed to help patients better manage their health with the intention to improve health outcomes, health care communication, and reduce costs [4,5]. With access to lab results and health indicators such as weight, blood pressure, and cholesterol, patients can promote early intervention when they encounter a deviation or problem or monitor improvements if following a new medication, exercise, or diet regimen [6]. Patients can also access information from various providers and necessary medical histories during emergencies to improve care transition coordination [7]. Patient portals can be convenient for medication refills, scheduling appointments, and allowing patients to communicate asynchronously with providers [6,8]. Owing to consumer demand and US government incentives for health information technology expansion, the adoption and use of patient portals is on the rise [9].

Patient portals are a promising but understudied clinical tool particularly in aging populations [10]. As older adults are more likely to have multiple chronic conditions (MCC) and higher health care utilization [11,12], they are likely to benefit from patient portal use to manage their conditions and health care services [13,14]. Although older adults are the fastest growing users of the internet [15,16], a lag in patient portal adoption remains, particularly among the oldest, less affluent, and lower educated older adults [17,18]. Although older adults show interest in patient portals [19-21], adoption barriers and low utilization have been identified [22]. Older adults pinpoint technology discomfort, privacy and security concerns, and lack of relative advantages as primary reasons for not using patient portals [22,23].

Understanding older adult opinions about portal user interface (UI) and user experience (UX) may lead to improvements in the accessibility, acceptability, and adoption of patient portals among older adults with MCC. UI typically focuses on the visual *look* of the design, including elements related to color, font, and images. UX targets the overall experience related to usability, usefulness, function, credibility, and satisfaction with the technology [24]. There are UI design recommendations for older users [25]; however, little is known about portal use and UX among older adults with MCC [26]. For example, portal email communication, lab results access, and electronic refill capabilities are important features for portal users [18,27], yet, it is unknown if these tools are commonly used or valued specifically by older adults with MCC. As older adults with MCC have much to gain from using portals, research is needed to better understand use behavior, perceived benefits, and strategies for increasing portal use among this specific population [26]. Therefore, the purpose of this study was to qualitatively explore perspectives from older adults with MCC regarding Kaiser Permanente Colorado’s (KPCO) established patient portal, *My Health Manager.* Framed by the Technology Acceptance Model (TAM), we qualitatively described the UI/UX, intent to use, and use behaviors among older Kaiser patients with MCC. Our specific research questions included the following: (1) How do participants use the portal?; (2) Why do participants use (or not use) the portal as they describe?; (3) How is the portal useful and usable?; and (4) How do these opinions and experiences influence participants’ intent to use the portal?

### Technology and Acceptance Model

The TAM is an information technology framework for understanding users’ adoption and use of emerging technologies particularly in the workplace environment and has been tested in older populations [28,29]. The theory posits that a person’s *intent to use* (acceptance of technology) and *usage behavior* (actual use) of a technology is predicated by the person’s perceptions of the specific technology’s *usefulness* (benefit from using the technology) and *ease of use*. Simply, users are more likely to adopt a new technology with high-quality UX design (ie, usable, useful, desirable, and credible). The TAM also suggests that perceptions of usefulness and ease of use are mediated by *external variables* including individual differences, system characteristics, social influences, and facilitating conditions.

### Kaiser Permanente Patient Portal: My Health Manager

*My Health Manager* (Table 1) provides personal health information related to patient diagnosis, prescriptions, laboratory results, and vaccination records. To improve provider-patient communication, *My Health Manager* offers features for patients to email providers and schedule appointments. Health management features that are designed to foster healthy eating and exercise habits incorporate personalized assessments and health self-management tools.

**Table 1:** Patient portal (My Health Manager) features summary.

Feature	Function
Appointment Center	Patients can schedule or cancel appointments
My Medical Record	Patients can view test results, immunization records, medical problem list, and care plans
Pharmacy Center	Patients can manage prescriptions and order medications
Health Guides and Health Management Tools	Access to health resources and self-management tools for diet, exercise, smoking cessation, and disease specific care
Message Center	Patients can email with their provider
Recently added features	e-visit and provider *chat* functions for nonemergent questions and visits

## Methods

This is an exploratory, descriptive qualitative study based on data collected from a series of focus groups [30,31]. This method is primarily used to better understand the needs and desired outcomes from a particular group of people [32]. As such, this study employed an exploratory, descriptive approach to describe UI and UX preferences, intent to use, and use behaviors of KPCO’s *My Health Manager* of older patients with MCC *.* All procedures were approved by the KPCO Institutional Review Board.

### Sample and Recruitment

We identified KPCO patients meeting the following inclusion criteria: aged ≥65 years, KPCO member for ≥1 year, presence of MCC (Charlson Comorbidity Index >2), and connected to 1 of 3 clinics in the Denver metro area with large geriatric patient populations. Non-English–speaking patients, individuals residing in skilled nursing facilities, and patients with a diagnosis of dementia were excluded. We then randomly selected potential participants stratified by age group (65 to 75 years; 76 to 85 years; and 86+ years) and portal user status (nonusers and users) to ensure participation from older participants and maximize the variability of experience with the portal. *Nonusers* were patients not registered for the portal, and *users* were those registered for the portal and logged into the portal within the last 6 months. Recruitment letters were mailed to 225 potential participants summarizing the study and providing an opt-out phone number to call if disinterested. A total of 210 potential participants (n=90 users and n=120 nonusers), who did not initially opt-out, were contacted via phone and invited to participate in focus groups. Recruitment resulted in an 18% acceptance rate (n=19 users and n=18 nonusers). Of the 37 patients that were scheduled to attend focus groups, 24 patients (n=15 users; n=9 nonusers) participated. We contacted the 13 participants who did not show up for their scheduled focus group to reschedule: 2 patients were unable to reschedule and 11 were lost to follow-up.

### Focus Groups and Question Guide

We conducted 6 focus groups (3 nonuser groups and 3 user groups) lasting approximately 90 min at the KPCO facility most convenient for the participant. Around 3 to 6 patients participated in each group. Focus groups were semistructured in format, allowing for probing and extended discussion on topics of interest to the participants. Before group discussion, participants were asked to complete a demographics and technology utilization survey. The survey collected information regarding income, education, cell phone, email, internet, digital communication, and social media use. Other demographic variables including race/ethnicity, age, and days since last portal log-in were captured from KPCO’s electronic medical record during sampling procedures. Participants were then asked questions related to *My Health Manager*. In addition to a traditional question answer session, patients were asked to *think aloud* [33] as a group, whereas the interviewer navigated a mock *My Health Manager* portal (Textboxes 1 and 2). This method has previously been used to assess health literacy and numeracy of patient portals among patients over 65 years [34]. Focus groups were audio-recorded for accuracy in data.

### Data Analysis

A theoretically driven approach [35] based on the TAM was used for analysis to capture participants’ opinions and experiences. The analysis was completed by 2 female doctoral-level researchers: JDP, a social work assistant professor with prior qualitative experience, and KG, a research assistant new to qualitative approaches. Audio files were first professionally transcribed verbatim. The unit of analysis, defined as a completion of 1 thought, was determined by the analytic team. Units of analysis ranged from a brief 3-word sentence to a paragraph of 6 sentences and were entered into Microsoft Excel to ensure that the units were consistent across coders. For initial coding, coders used a TAM-based theoretically driven code book developed *a priori* to code the units. The codebook included a list of TAM codes (eg, TAM constructs related to user intent, usage behavior, usefulness, and ease of use), code meaning, and criteria for using each code to capture participants’ perceptions of their intent to use and UX of *My Health Manager*. To prevent coders from forcing the units to fit with the TAM framework, coders used a *no code* option for responses that did not meet the code meaning and criteria for the *a priori* codes.

**Overview of focus group questions and portal features for users.**

Preliminary questions:

- Why did you enroll in My Health Manager?
- What features do you use most?

Think Aloud Questions:

- As we navigate this feature, what do you think about it?
- Why do you use it?
- How would you improve this feature?
- What helps you use this feature?
- What outcome do you want to achieve by using the feature?

**Overview of focus group questions and portal features for nonusers.**

Preliminary questions:

- Are you interested in using My Health Manager?
- Are there reasons why you do not use My Health Manager?

Think Aloud Questions:

- As we navigate the portal, are there features you might like to use?
- What do you like about this feature? Or What do you dislike about this feature?
- Why would you want to use these features? Or Why would you prefer to NOT use these features?
- What supports would you need to use this feature?
- How do you currently accomplish this task (related to feature)?

However, to ensure that participants’ thoughts were fully captured, coders also used a combination of *open* and *in vivo* coding (the use of participants’ own words as a code) to add inductive codes to the code book as needed. Inter-rater reliability was calculated for all transcripts (*K*=0.98), reflecting adequate consistency in coding across coders. Patterned coding was then employed on initial codes to identify (1) patterns in responses between users and nonusers and (b) patterns in responses between ease of use, perceived usefulness, intent to use, and UX. These patterns were then used to form themes related to each research question and develop overall findings. The analytics team met regularly through the analytic process to discuss codes and correct any disagreements in coding and thematic findings.

## Results

### Participants

Participants (N=24) were of a mean age of 78 years and were primarily white women (Table 2). Patient portal users had logged in to *My Health Manager* on an average 17.1 (SD 28.3) days before recruitment. All but one participant used a cell phone regularly, primarily a mobile phone. The majority of participants, regardless of user status, used email and looked up information on the Web. Approximately half of the participants used social media, played video games, and used video chat, whereas instant messaging was less popular.

### Technology Acceptance Model Description for My Health Manager

**Figure 1:**
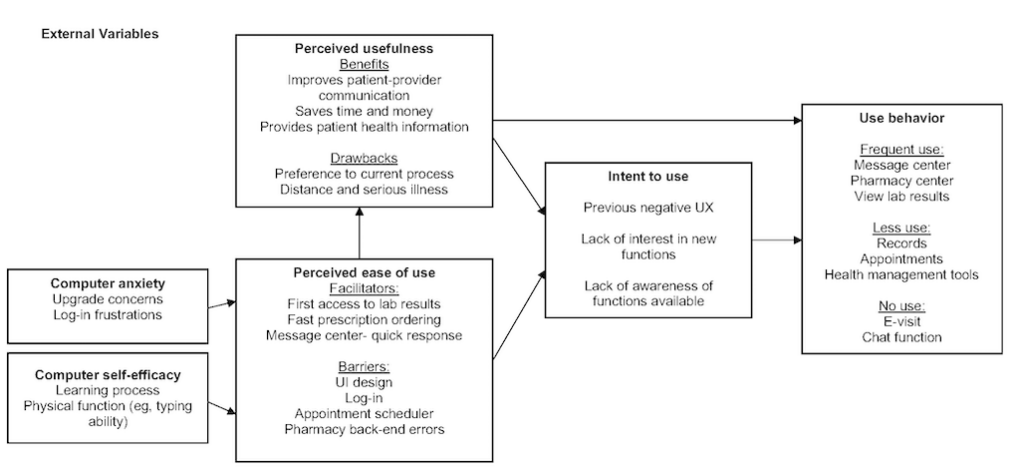
Technology Acceptance Model description for My Health Manager. UX: user experience; UI: user interface.

On the basis of the TAM, Figure 1 illustrates the findings from the focus groups regarding UI and UX, intent to use, and use behavior for My Health Manager.

#### Use Behavior

Portal users described their use of various *My Health Manager* features (listed in Table 1). The email Message Center was the most popular feature used by *My Health Manager* users. Of the participants, 1 stated, “Yeah, I email my doctors a lot!” whereas another stated, “And I like text chatting with the sending an e-mail to my doctors [feature]; just to ask a question”. The Pharmacy Center was also commonly used to refill medications. For example, a participant said, “I use the pharmacy part every time. I hardly ever call in the pharmacy any more”. Viewing lab results in the My Medical Record page was frequently used. As 1 participant noted, “I especially like looking up the results of my test and finding out what those tests are for and if there [is] anything I need to be concerned about.”

Participants did not commonly use other My Medical Record features including viewing diagnosis list, care plans, or immunizations records. In terms of using the Appointment Center to schedule visits, 1 participant explained, “I really like using the website. I have made appointments and been shocked when I got them the next day on the computer. So sometimes I try to check there first, and then I’ll call if I feel like I need to come in and I can’t”. However, some participants unsuccessfully tried to use the Appointment Center and the majority of participants called the Kaiser phone line to schedule appointments. Only 1 participant stated they used the health guides and health management tools: “I think that’s a pretty neat feature. And then I’ve looked up things for my friends when they have questions.” None of the participants had used the newly added *My Health Manager* features including the e-visit or provider chat.

**Table 2:** Participant characteristics.

Characteristics		Users (N=15)	Nonusers (N=9)	Total (N=24)
Age (years), mean (SD)		76.4 (4.9)	82.7 (3.4)	78.41 (5.4)
Female, n (%)		12 (80)	5 (56)	17 (71)
White, n (%)		12 (80)	7 (78)	19 (79)
Hispanic, n (%)		1 (7)	2 (11)	3 (13)
Days since *My Health Manager* log in, mean (SD)		17.1 (28)	—^a^	—
**Education, n (%)**				
	High School Graduate	1 (7)	5 (56)	6 (25)
	Some College Graduate	7 (47)	2 (22)	9 (36)
	College Graduate	7 (47)	2 (22)	9 (36)
**Income (US$), n (%)**				
	<$30,000	2 (13)	2 (22)	4 (17)
	$30,000 to $49,999	7 (47)	6 (67)	13 (54)
	$50,000 to $74,999	2 (13)	0 (0)	2 (8)
	$75,000 and more	2 (13)	0 (0)	2 (8)
	Choose not to answer	2 (13)	1 (11)	3 (13)
**Own cell phone, n (%)**				
	Smartphone	12 (80)	5 (56)	17 (71)
	A regular or basic phone	12 (80)	3 (33)	6 (25)
	Does not have a cell phone	3 (20)	1 (11)	1 (4)
**Technology utilization, n (%)**				
	Email	15 (100)	7 (78)	22 (92)
	Look up information on the Web	15 (100)	6 (67)	21 (88)
	Use social media	8 (53)	5 (56)	13 (54)
	Play computer games	12 (80)	3 (33)	15 (63)
	Video chat	7 (47)	4 (44)	11 (46)
	Instant messaging	6 (40)	2 (22)	8 (33)

^a^Not applicable.

#### Perceived Ease of Use

Although participants stated that the website was “pretty easy to use,” both portal user and nonuser participants (as shown during the focus group) were relatively negative about the UI and UX of *My Health Manager* (Textbox 3). Nonusers quickly identified UI design problems related to font size and colors while viewing the mock portal. In addition to some design issues, portal users noted challenges with using the portal or follow-up from using the portal system specifically related to registering with the system, logging in, and scheduling appointments. Several challenges were related to the back end of the system. For example, it was easy for patients to order their prescriptions on the Web, but in some cases, there were problems when participants went to the clinic to pick up the prescription. Participants who tried to use the appointment center said that they could not figure out how to schedule a visit on the portal. Those that were able to schedule an appointment on the portal experienced back-end problems at the clinic when they arrived to check-in for the visit. Despite some challenges in using the portal, many users thought *My Health Manager* was easy to use, and in some cases, *My Health Manager* was easier to use than more traditional services.

Perceived ease of use was impacted by participants’ level of computer anxiety and computer self-efficacy.

#### Computer Anxiety

Both portal users and nonusers expressed issues of computer anxiety, defined as apprehension or even fear of using the technology, which led to specific difficulties in using *My Health Manager*. As 1 user stated, “Well, I have just a couple of general thoughts about the Kaiser website. One is that what we all hate is instant upgrades, right? You hear the word ‘upgrade’ and you shudder.” Users worried that with every update, at some point they would be unable to use *My Health Manager* and that each new upgraded version created new use challenges. For example, during an upgrade, the log in page was modified, making it difficult for users to find the username and password text box that had previously been easy to use. The participant explained, “So recently—I don’t know how recently—but Kaiser upgraded, and all of a sudden, I couldn’t figure out how to log in. Now how stupid is that? So eventually, you know, I scanned and the ‘oh, there’s my name in the little arrow,’ but I thought, ‘couldn't they have warned us?’ That was my first thought.”

Therefore, after an upgrade, users were apprehensive about using the new version and typically found the new version to be more difficult to use than the previous version.

#### Computer Self-Efficacy

Users and nonusers both indicated that they were pretty confident in their ability to “figure things out” on the portal and felt good when they learned how to use a new *My Health Manager* feature or technology in general. However, participants discussed that although confident in their ability to use the portal, the process of learning how to use the website was challenging. A user stated:

Me and computers have problems anyway. It's like, ugh. Probably just inexperience of using the computer. I mean, I use the things that they have here, but it's not anything like going onto the website and that kind of stuff. But it was probably just not knowing where I was going or what I was doing.

Responses suggested that older participants believed they could use *My Health Manager* but acknowledged there would be a steep learning curve. Although learning to use the portal presented difficulties, some participants explained they simply did not have the ability to use specific features. One nonuser, who was interested in learning more about the provider email function, stated they could not use the new provider chat function because they could not type or respond quickly enough to instant messages with a provider. They explained, “You have to type in your chat...It’s fast...So email would be better.”

#### Perceived Usefulness

Participants described benefits and drawbacks regarding My Health Manager (Textbox 4).

**Perceived ease of use of My Health Manager.**

Participant quotes:

- Barriers

- User interface design

It’s hard for me to see.

The big print I can read. But I have to strain to see the small prints.

It’s like it’s not dark enough. Is that what other people say, too?

- Logging-in

My frustration is, it does not matter what I try to use, it never works. I can do this, this, this, this, this just exactly the way I remember the way I’m supposed to do it, and then you get down and it does not work. Enter password, wrong. This or that, something is wrong.

- Scheduling appointments

Nope, I couldn’t make it work...Because you don’t know what’s available. Does (the doctor) have a slot at 2:00 on such-and-such a day? They don’t tell you that... Step 7? Why is there seven steps just to make an appointment?

- Back-end errors

I don’t want to use (the Appointment Center) because there’s confusion among the people here for instance. And I’ll tell you, you make (an appointment), for instance I have sun damage because I'm out in the sun a lot, so I made an appointment to have it checked. And so that was the appointment. When I got here, to see the doctor, the nurse says, “Well, you have to see a PA.” I said, “Well, I just made an appointment on the website. I made the appointment, I wrote it down.”

Well, yeah. They went through a period of time, I think, when they were changing over which that’s really the only problem I’ve ever had with them. And they were significant because medications disappeared. I mean you order them, they were there. But other than that, it works very well. They straightened it out I think.

- Facilitators

- Simple, quick, and easy to use

It’s nice to be able to see the results and that stuff because that’s easy. Or if the doctor sends you a message. It’s easy to pick it up.

I have sent emails to my doc, especially when I don't want to come in and usually I get an answer within a very short time.

The test results are pretty easy to get. I really like it. I like the fact that I can graph my test results as opposed to just seeing the numbers.

**Perceived usefulness of My Health Manager.**

Participant quotes:

- Benefits of using *My Health Manager*

- Improves patient-provider communication

I love the fact that I can communicate with the doctor or any of the other doctors. I get complete descriptions on blood work and what happens with that.

You can as you are typing (via email feature), you can think and maybe, “No, that isn’t really what I want to say” instead of stumbling around. And you can do it more precise...Yeah, more organized. And then before you send it.

For me, it was an easy way to get non-emergent information to the doctor. For me, that's the easiest part of it is I can send stuff and they’ll either answer me or give me a call, one of the two.

- Saves time and money

And that makes sense, because all it is, is you don’t have to come in for that visit, which, if it’s difficult for you to get out, if the weather’s crappy and stuff like that.

When you initially send the e-mail to your doctor, sometimes they get back to you and they’ll say, “We’ll have a conference call. I’ve arranged a conference call to talk to you about it.” And that really saves a lot of time.

And it could be something just little or a prescription change or something to that effect that you really don’t have to come in and see the doctor about. And it’s more an efficient way of really the whole system working.

- Provides patient health information

I am an advocator for people taking control of their own health care versus relying on – that’s not to say I’m going to self-medicate or anything. But I believe in being well-informed about my healthcare and presenting options to my doctors and that sort of thing. So I like to be really informed about what’s going on.

But yeah, they don’t have any trouble because it’s nice that I can get messages from my doctors, telling me where I’m at. Or if I’ve had a blood test, I know that it’s okay or if it’s not

- Drawbacks of using *My Health Manager*

- Preference for current process

That’s hard. There’s yes and then no, because I don’t hardly call into the hospital or I know when my appointments are and when I come, they tell me to call in to get my medicine. I don’t because I live so close, it’s even a little walk for me to come and pick them up. So, the way I feel now, I can still do things like I’m doing now. I mean you have to walk four or five blocks to come down here, and then I take what I can do by myself. And so, then sometimes I meet people here that I know, and for me it’s just like getting out for a little trip.

- Distance and serious illness

I think if I had more in my body or that I had more problems that (using My Health Manager) would be good. But I am never sick. Of course, you never want something to happen. I don’t go to my doctor real often either

If I lived far, it would work very good for me.

#### Benefits

Portal users expressed a clear benefit in using *My Health Manager*, in general and for very specific features. Generally speaking, users expressed that *My Health Manager* was useful in communicating with their provider, accessing health information, saving time and money, and addressing health concerns without a clinic visit.

#### Drawbacks

On the contrary, nonusers stated they preferred to accomplish health-related tasks using their current process and indicated the portal would be more helpful for particular people: those living at a distance and those with serious illness. Nonusers preferred to use the telephone or clinic pharmacy for prescriptions, seeing their provider in person when asking nonemergent questions and calling to make clinical appointments. However, it is important to note that participants described alternative benefits for their current methods. Although portal users thought using *My Health Manager* for prescription refills was useful in getting medications, nonusers liked going to the pharmacy in person because it got them out of the house and kept them active. Nonusers acknowledged that *My Health Manager* would be useful for patients *far away* from their providers and *sicker* patients. Nonusers understood why people would want to use the system. It simply did not seem useful to them personally.

##### Intent to Use

Participants’ intention to continue using or start using *My Health Manager* was influenced by their perceived ease of use and perceived usefulness.

##### Previous Negative User Experience

Once a participant had a negative UI with *My Health Manager* or a specific feature, they had little interest in trying again. For example, a nonuser who tried to register for *My Health Manager* was so frustrated with their registration experience that he/she did not want to try again, and gave the following explanation:

I tried to get on [My Health Manager] several years ago and that was when they were sending the password by mail. I lost the password, and I forgot that you had to have (a password). I forgot all that. I again tried to get on it and didn’t have a password so I thought, “Well, I’ll just start over again.” It wouldn’t let me, so I said, “Well, the heck with it then.

Also, as stated above, users expressed many challenges in using the Appointment Center; therefore, participants showed little intent to use the Appointment Center until the *glitches* were fixed.

##### Lack of Interest in New Functions

Nonusers and users alike were not interested in using the recently added features of e-visits and chat functions. Few participants saw the value in using these features, articulating comments such as, “But I wouldn’t use it because I don’t see any need to, personally. I’m not saying other people wouldn’t.” Nonusers wanted to continue seeing their providers in person or talking on the phone, and users wanted to continue using the portal as is. A participant explained about possibly using the e-visit feature:

Usually, if I want to see the doctor, I want to see the doctor. And I know what it is why I’m going, and what it is I want to talk to them about. If it’s just real simple, I can just e-mail him or call him. If I want to see my doctor, I undoubtedly have to make an appointment to see him. And if I want to see my doctor, I want to go see my doctor.

##### Lack of Awareness of Functions Available

Intent to use was also influenced by participants’ awareness of *My Health Manager* features and access to help using the website. Most participants did not know about the new features, and nonusers did not know about the basic features available via *My Health Manager* in general. As 1 nonuser participant stated, “You can see how I read on the computer, because I’d never seen that—make a—schedule an appointment. That wouldn’t occur to me”; participants did not know what features were available or how to use them.

## Discussion

### Principal Findings

This study supports the growing literature suggesting many older patients, including those with MCC, are interested in using and are already using patient portals to help manage their health [22]. This is also the first study to use the TAM to qualitatively explore the connections between perceived usefulness, ease of use, and intent to use for a patient portal among older patients with MCC.

The TAM framework and supporting evidence [17,18] indicates several external variables influencing perceived ease of use with patient portals. Our study participants identified only 2 external variables: computer self-efficacy and anxiety. Specific patient portal user trainings offered in-person and/or on the Web may help older adults learn how to use the portal and when to use specific features [36]. Caregivers and family members are also helpful in reducing technology-specific anxiety [22,37], but more research is needed to inform portal design for shared access with caregivers [38].

Email, pharmacy, and medical lab result sections were popular and perceived as both useful and easy to use. This use behavior is consistent with other patient portal research in older populations [4,8]. These features are simple and quick while improving perceived patient-provider communication, satisfaction with access to health information, and fast medication management. Nonusers interested in the portal may be directed to these most popular, usable features. Research shows that once older adults are engaged in a technology, they tend to be high utilizers [23]. Therefore, promoting adoption of popular, easy-to-use features may foster patient satisfaction and further use of additional portal features. For example, promoting the email feature initially to encourage a patient to then try the portal pharmacy system.

Other features, particularly the Appointment Center, are difficult to use and do not offer perceived benefit to patients in this study. It is easier for patients to simply call to schedule appointments. There are also UI design issues related to small fonts and poor coloring, and negative UX influenced participants’ intent to use the portal. These results align with the TAM and previous work suggesting that technology acceptance is determined by the perceived value and degree of burden. Older adults are unlikely to adopt burdensome technologies. Therefore, health systems should obtain ongoing UI and UX feedback from older adults with MCC when developing new tools and updates. The Department of Veteran Affairs implemented an ongoing feedback strategy that fostered adoption of their patient portal [39].

In terms of perceived usefulness, participants in this study suggested that patients *far away* from their providers would particularly benefit from the patient portal. However, older adults in rural areas are less likely to use patient portals [40], and internet use is lower among people in rural settings, especially among people with MCC [41]. Low internet and health technology use in rural communities is often attributed to limited access and awareness [42]. Recent improvements to broadband access [43] in rural communities may lead to increased portal adoption. However, more research is needed to determine best strategies for promoting portal engagement among older adults with MCC living in rural settings.

Participants indicated the portal would be helpful for *sicker* patients. Although we did not follow up to acquire a better definition of *sicker*, older patients with serious illness, owing to complex care needs, may benefit from portal use. There is some evidence substantiating increased portal adoption among older adults with worse health status [44]. A few studies indicate that people with cancer have positive perceptions of patient portals [45,46]. Although older adults with serious illness may be a target population for portal adoption, little is known about patient portal utility for patients with advanced or serious illness.

Preference for current methods is also a drawback to perceived usefulness and barrier to patients’ intent to use. Participants value going to the clinic or pharmacy for physical activity and social engagement. With these values in mind, portal designers should consider adding functions that encourage older patients to *get out of the house* and connect personally with their providers. As portals advance, it is important to also respect the patients’ need for a face-to-face connection with their providers. However, providers may consider using some face-to-face and phone-based time to encourage portal use [21]. Face-to-face and phone-based encouragement (eg, “Did you know you can schedule your next appointment in the convenience of your home online? Just go to My Health Manager”) from providers and staff may increase patient awareness of beneficial features. In this study, health management tools and newer features were not used primarily owing to a lack of awareness.

In this sample, patient portals are not preferred by everyone, and other older adults with MCC may feel similarly. Usage varies greatly: some patients will never use the portal, other current users will continue to use only a few features, whereas another group will use every available option. Explicit nonusers appear to prefer human and face-to-face contact, which has previously been reported from a diverse sample of Kaiser patients [47]. Regardless of preference, technology-based health care interactions are increasing, and portal use may be expected. Addressing UI and UX challenges and promoting perceived benefits (improving commination, saving time, and access to personal information) may improve the intent to use patient portals among older adults with MCC.

### Limitations

Although our study is an in-depth analysis of perceptions of older adults with MCC of a specific patient portal, the use behaviors and experience may differ across portal systems. While employing a descriptive qualitative approach for understanding portal UI and UX, intent to use, and use behavior of My Health Manager, we used only focus groups for data collection. This work would have benefited from inclusion of other data sources such as observation. We were unable to recruit as many portal nonusers resulting in limitations of our nonuser feedback. Although we recruited participants representing a wide age range, we did not maintain equal participation from each age group. The sample was also relatively well educated, middle income, and technology users, lacking specific input from underprivileged populations with less access to technology resources.

### Conclusions

Older adults are interested in using patient portals and are already taking advantage of the features available to them. We have the opportunity to better engage older adults to use portals but need to pay close attention to key considerations promoting usefulness and ease of use. We recommend implementing portal user trainings, family and caregiver support, ongoing user feedback, and provider encouragement to improve intent to use and adoption among older adults with MCC.

## Abbreviations

MCC: multiple chronic conditions
KPCO: Kaiser Permanente Colorado
TAM: Technology Acceptance Model
UI: user interface
UX: user experience

## References

[1] ONC: Office of the National Coordinator for Health Information Technology.

[2] ONC: Office of the National Coordinator for Health Information Technology.

[3] Coughlin SS, Prochaska JJ, Williams LB, Besenyi GM, Heboyan V, Goggans DS, Yoo W, de Leo G (2017). Patient web portals, disease management, and primary prevention. Risk Manag Healthc Policy.

[4] Ford EW, Hesse BW, Huerta TR (2016). Personal health record use in the United States: forecasting future adoption levels. J Med Internet Res.

[5] Tenforde M, Jain A, Hickner J (2011). The value of personal health records for chronic disease management: what do we know?. Fam Med.

[6] Kruse CS, Argueta DA, Lopez L, Nair A (2015). Patient and provider attitudes toward the use of patient portals for the management of chronic disease: a systematic review. J Med Internet Res.

[7] Bouri N, Ravi S (2014). Going mobile: how mobile personal health records can improve health care during emergencies. JMIR Mhealth Uhealth.

[8] Zarcadoolas C, Vaughon WL, Czaja SJ, Levy J, Rockoff ML (2013). Consumers' perceptions of patient-accessible electronic medical records. J Med Internet Res.

[9] Frost and Sullivan (2018). Trade NAVI.

[10] Ammenwerth E, Schnell-Inderst P, Hoerbst A (2012). The impact of electronic patient portals on patient care: a systematic review of controlled trials. J Med Internet Res.

[11] (2018). Centers for Disease Control and Prevention.

[12] (2016). Federal Interagency Forum on Aging Related Statistics.

[13] Gee PM, Greenwood DA, Paterniti DA, Ward D, Miller LM (2015). The eHealth Enhanced Chronic Care Model: a theory derivation approach. J Med Internet Res.

[14] Smith SG, Pandit A, Rush SR, Wolf MS, Simon C (2015). The association between patient activation and accessing online health information: results from a national survey of US adults. Health Expect.

[15] (2018). Pew Research Center.

[16] (2018). Pew Research Center.

[17] Smith SG, O'Conor R, Aitken W, Curtis LM, Wolf MS, Goel MS (2015). Disparities in registration and use of an online patient portal among older adults: findings from the LitCog cohort. J Am Med Inform Assoc.

[18] Gordon NP, Hornbrook MC (2016). Differences in access to and preferences for using patient portals and other eHealth technologies based on race, ethnicity, and age: a database and survey study of seniors in a large health plan. J Med Internet Res.

[19] Price MM, Pak R, Müller H, Stronge A (2012). Older adults’ perceptions of usefulness of personal health records. Univ Access Inf Soc.

[20] Nahm E, Sagherian K, Zhu S (2016). Use of patient portals in older adults: a comparison of three samples. Stud Health Technol Inform.

[21] Irizarry T, Shoemake J, Nilsen ML, Czaja S, Beach S, DeVito DA (2017). Patient portals as a tool for health care engagement: a mixed-method study of older adults with varying levels of health literacy and prior patient portal use. J Med Internet Res.

[22] Sakaguchi-Tang DK, Bosold AL, Choi YK, Turner AM (2017). Patient portal use and experience among older adults: systematic review. JMIR Med Inform.

[23] Wildenbos GA, Peute L, Jaspers M (2017). Facilitators and barriers of electronic health record patient portal adoption by older adults: a literature study. Stud Health Technol Inform.

[24] Canziba E (2018). Hands-On UX Design for Developers: Design, Prototype, and Implement Compelling User Experiences from Scratch.

[25] Fisk A, Rogers W, Charness N, Czaja S, Sharit J (2009). Designing for Older Adults: Principles and Creative Human Factors Approaches, Second Edition.

[26] Greenberg AJ, Falisi AL, Finney RL, Chou WS, Patel V, Moser RP, Hesse BW (2017). Access to electronic personal health records among patients with multiple chronic conditions: a secondary data analysis. J Med Internet Res.

[27] Redelmeier DA, Kraus NC (2018). Patterns in patient access and utilization of online medical records: analysis of MyChart. J Med Internet Res.

[28] Davis FD, Bagozzi RP, Warshaw PR (1989). User acceptance of computer technology: a comparison of two theoretical models. Manage Sci.

[29] Venkatesh V, Davis FD (2000). A theoretical extension of the technology acceptance model: Four longitudinal field studies. Management Science.

[30] Groves S, Burns N, Jennifer G (2019). The Practice Of Nursing Research: Appraisal, Synthesis, And Generation Of Evidence.

[31] Nayar S, Stanley M (2016). Qualitative Research Methodologies for Occupational Science and Therapy.

[32] Bradshaw C, Atkinson S, Doody O (2017). Employing a qualitative description approach in health care research. Glob Qual Nurs Res.

[33] Jaspers MW, Steen T, van den Bos C, Geenen M (2004). The think aloud method: a guide to user interface design. Int J Med Inform.

[34] Sharit J (2014). The roles of health literacy, numeracy, and graph literacy on the usability of the VA's personal health record by veterans. J Usability Stud.

[35] Saldana J (2009). The Coding Manual for Qualitative Researchers.

[36] Sieck CJ, Hefner JL, Schnierle J, Florian H, Agarwal A, Rundell K, McAlearney AS (2017). he rules of engagement: perspectives on secure messaging from experienced ambulatory patient portal users. JMIR Med Inform.

[37] Sarkar U, Bates DW (2014). Care partners and online patient portals. J Am Med Assoc.

[38] Latulipe C, Quandt SA, Melius KA, Bertoni A, Miller DP, Smith D, Arcury TA (2018). Insights into older adult patient concerns around the caregiver proxy portal use: qualitative interview study. J Med Internet Res.

[39] Nazi KM, Turvey CL, Klein DM, Hogan TP (2018). A decade of veteran voices: examining patient portal enhancements through the lens of user-centered design. J Med Internet Res.

[40] Arcury TA, Quandt SA, Sandberg JC, Miller DP, Latulipe C, Leng X, Talton JW, Melius KP, Smith A, Bertoni AG (2017). Patient portal utilization among ethnically diverse low income older adults: observational study. JMIR Med Inform.

[41] Wang J, Bennett K, Probst J (2011). Subdividing the digital divide: differences in internet access and use among rural residents with medical limitations. J Med Internet Res.

[42] Greenberg AJ, Haney D, Blake KD, Moser RP, Hesse BW (2018). Differences in access to and use of electronic personal health information between rural and urban residents in the United States. J Rural Health.

[43] (2016). Federal Communications Commission.

[44] Lober WB, Zierler B, Herbaugh A, Shinstrom SE, Stolyar A, Kim EH, Kim Y (2006). Barriers to the use of a personal health record by an elderly population. AMIA Annu Symp Proc.

[45] Zide M, Caswell K, Peterson E, Aberle DR, Bui AA, Arnold CW (2016). Consumers' patient portal preferences and health literacy: a survey using crowdsourcing. JMIR Res Protoc.

[46] Alpert JM, Morris BB, Thomson MD, Matin K, Brown RF (2018). Implications of patient portal transparency in oncology: qualitative interview study on the experiences of patients, oncologists, and medical informaticists. JMIR Cancer.

[47] Lyles CR, Allen JY, Poole D, Tieu L, Kanter MH, Garrido T (2016). "I Want to Keep the Personal Relationship With My Doctor": Understanding Barriers to Portal Use among African Americans and Latinos. J Med Internet Res.

